# The importance of complete and high-quality genome sequences in *Aspergillus niger* research

**DOI:** 10.3389/ffunb.2022.935993

**Published:** 2022-07-15

**Authors:** Valeria Ellena, Matthias G. Steiger

**Affiliations:** ^1^ Austrian Centre of Industrial Biotechnology (ACIB GmbH), Vienna, Austria; ^2^ Institute of Chemical, Environmental and Bioscience Engineering, Vienna University of Technology (TU Wien), Vienna, Austria

**Keywords:** enzymes, citric acid, industrial strains, centromeres, NUMTs, mtDNA, gene annotation

## Abstract

The possibility to sequence the entire genome of an organism revolutionized the fields of biology and biotechnology. The first genome sequence of the important filamentous fungus *Aspergillus niger* was obtained in 2007, 11 years after the release of the first eukaryotic genome sequence. From that moment, genomics of *A. niger* has seen major progresses, facilitated by the advances in the sequencing technologies and in the methodologies for gene function prediction. However, there are still challenges to face when trying to obtain complete genomes, equipped with all the repetitive sequences that they contain and without omitting the mitochondrial sequences.

The aim of this perspective article is to discuss the current status of *A. niger* genomics and draw attention to the open challenges that the fungal community should address to move research of this important fungus forward.

## Introduction

*Aspergillus niger* is a major industrial species belonging to the genus *Aspergillus*, section *Nigri*. First described in 1867 by Van Tieghem in the Annales des Sciences Naturelles (Van Tieghem, 1867), *A. niger* has been used for more than a century to produce citric acid, a bulk chemical employed in many commodity products, such as cleaning agents, food and beverages, pharmaceuticals and cosmetics (Currie, 1917; Baker and Bennett, 2007). Moreover, it is used to produce gluconic and fumaric acids (Baker and Bennett, 2007). Besides being a good organic acid producer, *A. niger* is also employed for the production of various enzymes, such as amylases, invertase, pectinases, phytases and proteinases. The wide range of temperature (6°C - 47°C) and pH (1.4 - 9.8) at which *A. niger* can grow allows it to be ubiquitous (Schuster et al., 2002). This species can cause allergic reaction and infectious diseases in patients with an impaired immune system (Schuster et al., 2002; Paulussen et al., 2017). Yet, being nonpathogenic and nontoxic for healthy individuals, with a long history of safe industrial use, many *A. niger* strains are classified as biosafety level 1 by the American Type Culture Collection (ATCC). Despite its century long use as industrial workhorse (Currie, 1917), its first genome sequence was only obtained in 2007 (Pel et al., 2007), more than a decade after the release of the first fungal genome sequence, belonging to the yeast *Saccharomyces cerevisiae* (Goffeau et al., 1996). The aim of this perspective article is to highlight recent developments in the field of genomics of *A. niger* and the future challenges that researchers have to address to improve our understanding of this important fungal species.

## Is the history of the *A. niger* genome sequences biased?

In 2007, the early ancestor of the enzyme-producing strains, CBS 513.88, was the first *A. niger* strain to be sequenced (Pel et al., 2007). The availability of this genome sequence allowed the identification of genes involved in a variety of cellular and metabolic processes, such as cell wall development, organic acid production, polysaccharide-degrading enzyme production and protein secretion (Pel et al., 2007). Genetic markers of the well-known flexible metabolism and nutritional versatility of this cell factory were identified in its genome sequence and its functionally annotated genes (Pel et al., 2007). Additionally, a complete set of apparently functional sex-related genes as well as several secondary metabolite gene clusters were identified (Pel et al., 2007). All in all, the 2007 publication provided new opportunities to study the biology of *A. niger* and to improve the current industrial strains, as confirmed by the high number of scientific papers (more than 800) which cited it since then.

Afterwards, genome sequencing of the citric acid producing strain ATCC 1015 in 2011 allowed to perform comparative analyses between the two sequenced strains and get important insights into their industrially relevant differences (Andersen et al., 2011).

Since the release of the first two *A. niger* genome sequences, the number of intra- and inter-species comparative genomic analyses increased (de Vries et al., 2017; Vesth et al., 2018), as well as the number of released *A. niger* genome sequences. At present, 24 A*. niger* genome sequences, including those of strains CBS 513.88 and ATCC 1015, are available at GenBank (RRID : SCR_002760, Benson et al., 2012) while the sequence of strain NRRL3 can be found at JGI´s MycoCosm (RRID : SCR_005312, Grigoriev et al., 2014) (**Table 1**). However, this is still a very limited number compared to the genome sequences available for other relevant-to-human aspergilli, such as *A. fumigatus* (more than 270 genomes deposited at GenBank) or *A. oryzae* (more than 100 genomes deposited at GenBank). Despite this small number, the scientific community studying *A. niger* is one of the largest fungal communities worldwide (Cairns et al., 2018). This imbalance might be explained by the interest of industrial companies in this relevant fungus which, on the one hand has been a stimulus to launch the first genome sequencing projects while on the other might have limited the diffusion of the obtained sequences. The first *A. niger* genome (CBS 513.88) was financed by the Dutch multinational company DSM and the genome of strain NRRL3 by the US based company Integrated Genomics (Baker, 2006). Moreover, most of the *A. niger* genome sequences deposited at GenBank belong either to industrial strains or to strains potentially relevant for industrial applications. However, the industrial involvement in the *A. niger* research also means that a number of genome sequences, those of the strains currently used as cell factories, are most likely kept confidential. This bias in the *A. niger* genome research towards industrially applied projects can limit other research fields, such as evolution and speciation, including the study of cryptic species, pathogenicity or adaptation to climate change, where sequences of environmental isolates are essential. Contributing to this effort, the genome sequence of the (non-industrial) neotype strain of *A. niger*, CBS 554.65, was made available in our publication of 2021 (Ellena et al., 2021). This neotype strain serves as reference strain for taxonomical and morphological analyses.

**Table 1:** Overview of the available *A. niger* genome sequences.

Strain	Assembly Accession	Submission Date	Sequencing Technology	Sequence Length (bp)	Number of scaffolds	Contig N50 (Mbp)	Contig L50	Related publication
CBS 513.88 *, #	GCA_000002855.2	11.04.07	BAC tiling	33,975,768	19	0.11	96	Pel et al., 2007
ATCC 1015 *	GCA_000230395.2; Updated annotation at MycoCosm (v4.0)	12.10.11	Shotgun	34,853,277	24	1.94	6	Andersen et al., 2011
NRRL3	Available at MycoCosm (link in the table caption)	01.04.14	Shotgun	35,245,396	15	2.81	5	Aguilar-Pontes et al., 2018
SH-2	GCA_000633045.1	17.04.14	Illumina GAII	34,604,268	349	0.30	40	Yin et al., 2014
An76	GCA_001515345.1	19.12.15	Illumina Hiseq	34,880,193	669	1.78	8	Gong et al., 2016
ATCC 10864	GCA_001715265.1	29.08.16	Illumina HiSeq	36,172,237	310	0.68	16	Paul et al., 2017
A1	GCA_001741885.1	21.09.16	Illumina MiSeq	34,635,689	319	0.13	88	Yin et al., 2017
H915-1	GCA_001741905.1	21.09.16	PacBio; Illumina MiSeq	35,881,929	25	4.44	4	Yin et al., 2017
L2	GCA_001741915.1	21.09.16	PacBio	36,413,165	28	4.16	4	Yin et al., 2017
JSC-093350089	GCA_001931795.1	04.01.17	Illumina HiSeq	36,079,011	223	0.22	52	Romsdahl et al., 2018
FGSC A1279	GCA_002740505.1	31.10.17	Illumina HiSeq	35,378,992	402	0.29	38	Wang et al., 2018
N402 (ATCC 64974) *	GCA_900248155.1	27.01.18	Illumina HiSeq; PacBio	35,570,168	19	2.87	4	Laothanachareon et al., 2018
CBS 101883	GCF_003184595.1	04.06.18	Illumina	35,853,610	107	0.41	28	Vesth et al., 2018
ATCC 13496 *	GCA_003344705.1, Improved assembly at MycoCosm (v2.0)	27.07.18	Illumina	35,690,699	133	0.37	30	Vesth et al., 2018
ATCC 1015D-2	GCA_002211485.2	15.11.18	PacBio; Illumina	35,806,243	25	3.44	5	Sichtig et al., 2019
MOD1-FUNGI2	GCA_004634315.1	04.04.19	Illumina NextSeq 500	33,793,555	3192	0.02	568	–
LDM3 *	GCA_009812365.1	31.12.19	PacBio; Illumina HiSeq	35,265,186	14	4.03	4	Sui et al., 2020
RAF106	GCA_011316255.1	16.03.20	PacBio Sequel	35,088,461	10	4.12	4	Fang et al., 2020
3.316	GCA_013618955.1	24.07.20	PacBio; Illumina	34,941,939	13	3.84	4	Ya-Nan et al., 2021
F3_4F2_F	GCA_015586215.1	18.11.20	Illumina NovaSeq	37,317,966	361	0.63	19	Blachowicz et al., 2021
F3_4F1_F	GCA_015586235.1	18.11.20	llumina NovaSeq	37,533,732	342	0.62	21	Blachowicz et al., 2021
F3_1F3_F	GCA_015586245.1	18.11.20	Illumina NovaSeq	35,865,776	75	0.65	20	Blachowicz et al., 2021
DSM 1957	GCA_016162265.1	21.12.20	Illumina	35,598,484	138	0.50	23	Detain et al., 2022
CBS 554.65	GCA_019288275.1	20.07.21	PacBio Sequel	40,425,233	17	4.07	4	Ellena et al., 2021
L14	GCA_019843515.1	01.09.21	Illumina HiSeq	36,095,484	25	4.20	3	–

Genomes with manually curated gene annotation are underlined. The genome of strain NRRL3 can be accessed at this link: https://mycocosm.jgi.doe.gov/Aspni_NRRL3_1/Aspni_NRRL3_1.home.html.

An asterisk (*) next to the strain name indicates that the genome sequence of the strain is present in FungiDB. #: reference genome.

## Centromeres and NUMTs are part of the genome – let´s include them!

The number of the released genomes increased hand in hand with the quality of these sequences. The advent of next-generation sequencing (NGS) technologies, such as those based on the Illumina or the PacBio sequencing platforms, represents a key advance in biology and genome research. NGS allows to obtain very high-quality whole genome assemblies at low costs and high speed. A comparison of the genome assembly statistics between the early *A. niger* genomes obtained by shotgun sequencing (CBS 513.88 and ATCC 1015) and more recent releases obtained by PacBio clearly shows the improved quality, in terms of coverage, number of scaffolds, N50 and L50 values (**Table 1**). Additionally, NGS makes it possible to cover highly repetitive regions of the genomes, which were difficult to capture by shotgun sequencing (Smith et al., 2012). This is for example the case of the centromeres which, due to their AT-rich repetitive sequence, are missing from earlier genome models. Centromeric sequences are essential for studying DNA-protein co-evolution and chromosome stability (Smith et al., 2012; Friedman and Freitag, 2017). In this respect, centromere research holds great potential for the development of novel antifungal agents interfering with the centromeres (Smith et al., 2012) or for the establishment of more genetically robust industrial strains (Friedman and Freitag, 2017).

Besides the centromeres and other difficult-to-sequence loci, other sequences might be missing from earlier genome projects. This is the case for NUMTs – nuclear sequences of mitochondrial origins – which designate stretches of DNA that were transferred from the mitochondrial to the nuclear genome. While some of these sequences are undetectable due to mutation or deletion events, others maintain high homology to the mitochondrial sequence (Richly, 2004; Hazkani-Covo et al., 2010). When depositing a new nuclear genome sequence to a public database, such sequences are automatically identified as mitochondrial contaminants and need to be removed from the assembly to proceed with the genome release. In the course of our research, we were notified of the presence of two putative mitochondrial contaminant sequences of 1,922 bp and 124 bp. In this case, we verified the nuclear localization of these sequences by read coverage, PCR and Sanger sequencing and the confirmed NUMTs were then included in the final assembly. In other genomes, however, such sequences might have been removed during the annotation process (Hazkani-Covo et al., 2010). A BLAST search (NCBI BLAST, RRID : SCR_004870, Altschul et al., 1990) of these two sequences against the *A. niger* whole-genome shotgun contigs database does not show any significant alignments with sequences of CBS 513.88 or ATCC 1015, suggesting that they might have been missed from their genome assembly. The inclusion of verified NUMTs in the deposited sequences is not only important to provide complete genome models but can also have interesting implications in the study of species evolution and speciation (Hazkani-Covo et al., 2010). It was shown that the evolutionary rate of fungal mtDNA is different from the one of nuclear DNA (Burger et al., 2003; Sandor et al., 2018). If this holds true for *A. niger*, comparison of NUMTs and their mitochondrial counterparts could provide new insights into the history of the species. Moreover, it would be reasonable to question whether NUMTs exert specific functions in the cell which might be interesting from an industrial viewpoint. For instance, connections between the respiratory chain function and the citric acid metabolism in *A. niger* have been discussed (Wallrath et al., 1991; Wallrath et al., 1992; Wang et al., 2015; Hou et al., 2018). It is therefore of interest to investigate if the mitochondrial genes encoding for electron transport proteins that can be identified in NUMTs are functional and play a role in metabolic pathways exploited in industrial processes. On the contrary, if these genes are not functional, one could investigate the reason why they are retained.

## Let´s not forget the mitochondrial genomes!

A major factor which slows down mitochondrial genome-based analyses in *A. niger* is the lack of mitochondrial genome sequences for most of the sequenced strains. It was previously observed that the number of total mitochondrial genomes deposited in GenBank has increased exponentially in the last decade (Smith, 2015). However, this is mainly true for animal species while protists, plants and fungi lag behind (Smith, 2015). Despite the increment of released nuclear genomes of *A. niger* strains in the past few years, only 3 A*. niger* mitochondrial sequences are available for this species, those of strains N909 (Juhász et al., 2004), CBS 513.88 (Pel et al., 2007) and CBS 554.65 (Ellena et al., 2021). The introduction of NGS technologies allows to obtain a large number of reads corresponding to the mitochondrial genome, without the need to isolate mitochondrial DNA. However, during the nuclear assembly process these sequence reads are typically sorted out and not included to assemble the mitochondrial genome. This is due to the fact that the read coverage of the mitochondrial genome is typically 10x higher compared to the nuclear genome (Ellena et al., 2021). In the absence of a mitochondrial sequence, the genetic content of a strain is only partially characterized (Joardar et al., 2012; Hugaboom et al., 2021). Besides providing unique opportunities to study evolution, phylogenetic relationships and intra-specific variations, mitochondrial DNA exerts important functions in a variety of cellular and metabolic processes. In addition to their role in the energy metabolism, mitochondrial genes are associated with virulence and pathogenicity, cell quiescence during adverse environmental conditions and drug resistance (Chatre and Ricchetti, 2014; Calderone et al., 2015). For instance, the resistance of strain N909 to oligomycin is linked to mutations in the mitochondrially encoded genes (Ward et al., 1986; Niedzwiecka et al., 2018). The availability of mitochondrial genome sequences can provide significant insights into a variety of processes, including metabolic pathways and disease control mechanisms. Thus, there is a clear need to re-evaluate existing genome projects and assemble and annotate the missing mitochondrial genomes.

## Which gaps in data integration and gene annotation are still to be filled?

Despite the incompleteness of the majority of the deposited *A. niger* genomes, genomics (and other omics) datasets are rapidly generated and accumulate in different online databases. Efficient systems are needed not only to securely store such data, but also to allow their accessibility by the research community. Data stored in different databases (such as MycoCosm, GenBank or ENA) or reported only in the literature should be integrated and rendered easy to search by the different research groups (Meyer et al., 2020). Moreover, digital tools should be implemented to support the analysis of this vast amount of data (Meyer et al., 2020). Very comprehensive overviews of the challenges faced to improve the accessibility and analysis of fungal omics data were previously published (Meyer et al., 2016; Meyer et al., 2020).

Furthermore, efforts should be made to align gene annotation between genome sequences of different strains and of different versions of the same strain. An initial version of the ATCC 1015 genome (v3.0 from MycoCosm) had different gene identifiers than a subsequent version (v4.0 from MycoCosm) of it. Although the annotation of the updated version was improved compared to version 3, not all available online tools adopted it. As a result, to effectively launch a gene search on different online tools, different gene identifiers are required. For example, when one wants to find some information about the *xlnR* transcription factor in FungiDB (RRID : SCR_006013, Basenko et al., 2018), they will have to search it in the format ASPNIDRAFT2_1183692 (gene identifier of v4.0), while when one wants to design a gRNA targeting this gene, they will need to input its old identifier from version 3 (48811-mRNA-T) in the CHOPCHOP (RRID : SCR_015723, Montague et al., 2014) search window. Therefore, the lack of integration of gene annotation between different resources and tools makes research unnecessarily more complicated and slower. To this regard, scientists are urged to carefully report not only the accession bank of the assembly they work with but also the version of the genome and any annotation modifications associated to it.

Besides the inconvenience of naming the same gene with different identifiers, gene annotation should also give some information about the function of the genes. Recently, Schäpe and colleagues were able to predict the biological processes of around 65% of the *A. niger* predicted genes by generating and interrogating gene co-expression networks (Schäpe et al., 2019). This represents an important advance in gene functional predictions, considering that previously only 40-50% of the genes were functionally predicted (Meyer et al., 2016; Cairns et al., 2018), and paves the way for a more comprehensive functional annotation of the *A. niger* genomes (Schäpe et al., 2019; Meyer et al., 2020).

## Concluding remarks

The recent increase of *A. niger* high-quality genome sequences has contributed to important developments in the study of this fungus and its industrial applications. Still, knowledge gaps exist. The first *A. niger* genome to be sequenced, the one of strain CBS 513.88, is still utilized as reference genome for *A. niger.* Its role as reference lies more on its manually curated annotation, than on the quality of the assembly itself, which, as mentioned before, lacks important regions, such as the centromeres. Therefore, caution should be taken when using this genome as a reference for new and, most likely, higher-quality sequences. In order to have a genome reference characterized by the high-quality standards of the current sequencing methods, the reference strain needs to be re-sequenced and updated with a novel NGS technology.

Complete and reliable genome models, consistent and up-to-date gene annotation, data integration between digital platforms and published literature, and data accessibility are the basis of good and successful science, but they are currently only partially achieved. This is severely hampering the progress in the *A. niger* research and highlights the urgent need of establishing a consolidated and common platform where the available datasets can be easily accessed. A similar platform, AspGD (Arnaud et al., 2010), was recently closed down because of funding issues. The information present on AspGD was transferred to FungiDB. FungiDB is an effective resource to compare different fungal species, however it lacks integrative information on single species level and literature is not regularly integrated. A community-based repository like the Saccharomyces Genome Database, SGD (RRID : SCR_004694, Cherry et al., 2012), provides a good example on how genome data of a single species can be effectively integrated and made accessible to researchers. A collaborative effort should be therefore undertaken by the fungal community studying *A. niger* to optimize the integration of the available genomic data and their regular update. This would greatly improve the analysis of such data and *A. niger* research, leading to potential interesting implications on a wide range of scientific relevant questions.

## Data availability statement

The datasets presented in this study can be found in online repositories. The names of the repository/repositories and accession number(s) can be found in the article/supplementary material.

## Author contributions

VE and MGS conceived the manuscript. VE wrote the manuscript and MGS provided critical feedback and input. All authors read and approved the final manuscript.

## Funding

The COMET center: acib: Next Generation Bioproduction is funded by BMK, BMDW, SFG, Standortagentur Tirol, Government of Lower Austria und Vienna Business Agency in the framework of COMET-Competence Centers for Excellent Technologies. The COMET-Funding Program is managed by the Austrian Research Promotion Agency FFG.

## Conflict of interest

Authors VE and MS were employed by Austrian Centre of Industrial Biotechnology (ACIB GmbH).

## Publisher’s note

All claims expressed in this article are solely those of the authors and do not necessarily represent those of their affiliated organizations, or those of the publisher, the editors and the reviewers. Any product that may be evaluated in this article, or claim that may be made by its manufacturer, is not guaranteed or endorsed by the publisher.
